# Intratumoral administration of the antisecretory peptide AF16 cures murine gliomas and modulates macrophage functions

**DOI:** 10.1038/s41598-022-08618-x

**Published:** 2022-03-17

**Authors:** Jan Kopecky, Julio Enríquez Pérez, Håkan Eriksson, Edward Visse, Peter Siesjö, Anna Darabi

**Affiliations:** 1 Glioma Immunotherapy Group, Division of Neurosurgery, Department of Clinical Sciences Lund, Faculty of Medicine, Lund University, Barngatan 4, 221 85 Lund, Sweden; 2 Section of Neurosurgery, Department of Clinical Sciences Lund, Skåne University Hospital, Lund, Sweden; 3 Department of Biomedical Science, Faculty of Health and Society, Malmö University, Malmö, Sweden

**Keywords:** Cancer, Cancer microenvironment, CNS cancer, Tumour immunology

## Abstract

Glioblastoma has remained the deadliest primary brain tumor while its current therapy offers only modest survival prolongation. Immunotherapy has failed to record notable benefits in routine glioblastoma treatment. Conventionally, immunotherapy relies on T cells as tumor-killing agents; however, T cells are outnumbered by macrophages in glioblastoma microenvironment. In this study, we explore the effect of AF16, a peptide from the endogenous antisecretory factor protein, on the survival of glioma-bearing mice, the tumor size, and characteristics of the tumor microenvironment with specific focus on macrophages. We elucidate the effect of AF16 on the inflammation-related secretome of human and murine macrophages, as well as human glioblastoma cells. In our results, AF16 alone and in combination with temozolomide leads to cure in immunocompetent mice with orthotopic GL261 gliomas, as well as prolonged survival in immunocompromised mice. We recorded decreased tumor size and changes in infiltration of macrophages and T cells in the murine glioma microenvironment. Human and murine macrophages increased expression of proinflammatory markers in response to AF16 treatment and the same effect was seen in human primary glioblastoma cells. In summary, we present AF16 as an immunomodulatory factor stimulating pro-inflammatory macrophages with a potential to be implemented in glioblastoma treatment protocols.

## Introduction

Glioblastoma is a Grade IV glioma and the most common primary malignant brain tumor, which, in addition to its high mortality rate and low median survival, is also resistant to all current conventional immunotherapy agents^1^. Therefore, the mainstay of glioblastoma therapy remains surgery with concomitant temozolomide (TMZ) and radiation followed by adjuvant TMZ. The immunological “coldness” of glioblastoma is largely explained by low neo-antigenicity, low levels of infiltrating T cells (i.e. principal agents in checkpoint inhibition) and overwhelmingly immunosuppressive tumor-associated macrophages (TAMs) in the glioblastoma tumor microenvironment (TME)^2^. Immunomodulating agents and more broadly, immunotherapy have been at the core of research of malignant tumors in the past years^3^, often recording remarkable successes in the treatment of other types of cancer^4–6^. While most of the agents approved for clinical use in cancer rely on direct T cell/NK cell- or indirect antibody/complement-mediated tumor killing^7,8^, a large volume of studies has focused on the role of the monocyte/macrophage system in immune evasion of tumor cells^9–11^. TAMs play an essential part in maintaining the tumor-promoting immunosuppressive microenvironment, as has been shown in several studies^12–14^. Macrophages as a cell type show fluidity in their phenotype in vivo but a simplified in vitro model has been broadly accepted—monocytes can differentiate to become naïve, uncommitted macrophages (designated M0) to then be further polarized along the M1 (proinflammatory, tumor-suppressive)—M2 (anti-inflammatory, tumorigenic) axis^15–18^. It is an appealing strategy to identify agents that would be capable of reducing the immunosuppressive factors secreted by TAMs and thus potentiate the antitumoral immune response.

AF16 is an active peptide of the larger antisecretory factor (AF) protein^19^. AF was discovered in the pituitary gland, cerebrum and intestinal mucosa but later found to be ubiquitously expressed by various tissues and immune cells, including macrophages^20–22^. Early on, studies described the anti-inflammatory effect of AF16 in a cholera-toxin colitis model^23^. When AF was blocked by monoclonal antibodies, T-cell mediated autoimmune encephalitis had worse outcomes in rats^24,25^. Moreover, AF16 was shown to reduce interstitial fluid pressure, edema and intracranial pressure after brain injury, autoimmune encephalitis, and in solid tumors^26–29^. A diet of AF-enriched egg yolk powder in xenografted glioblastoma models could improve survival, decrease tumor volumes and increase the effect of chemotherapy, tentatively by increasing uptake^30^. Importantly, exogenous AF administration has not been associated with any negative side effects^31^. However, the impact of AF on the immune aspect of the glioblastoma microenvironment in immunocompetent animals was not analyzed.

In this present study, we aim for the first time to investigate the functional effect of AF16 on glioblastoma survival in vivo and on the monocyte/macrophage population, with the emphasis on implications for the TME of glioblastoma. We also analyzed CD8^+^ T cells, M0 macrophages/microglia, and M1 and M2 macrophage populations in the glioblastoma TME, as well as selected inflammation-related molecules—cyclooxygenase 2 (COX-2) as a rate-limiting enzyme in the production of prostaglandins, e.g. PGE2 that is reportedly associated with glioblastoma immunosuppression and tumorigenesis^32^. Galectin 3 is associated with glioma progression and neo-angiogenesis but also with membrane damage, with galectin 3 being upregulated after temozolomide and radiotherapy treatment^33,34^. Phosphorylated Na^+^-K^+^-2Cl^−^ cotransporter isoform 1 (pNKCC-1) is an activated ion pump essential for regulating cell volume during e.g. apoptosis or glioma cell migration and it has been shown to upregulate after TMZ treatment in glioblastoma models^35^. We have previously proved that intratumoral administration of cytostatics is safe, offers better survival with less toxic effects compared to the systemic route, and gives rise to long-lasting immunological memory (depending on the drug and model)^36^. When combined with peripheral vaccination with tumor cells, the therapeutic effect from the intratumoral drug delivery is enhanced. Based on our previous promising results and to achieve high local drug concentrations while circumventing the blood–brain barrier in glioblastoma, AF16 and TMZ were administered intratumorally.

To our knowledge, such a comprehensive study of the immune effects of AF16 in glioblastoma has not been conducted to date.

## Results

### Intratumoral AF16 alone and combined with TMZ cured and improved survival of immunocompetent and immunocompromised glioma-bearing mice, respectively

**Figure 1:**
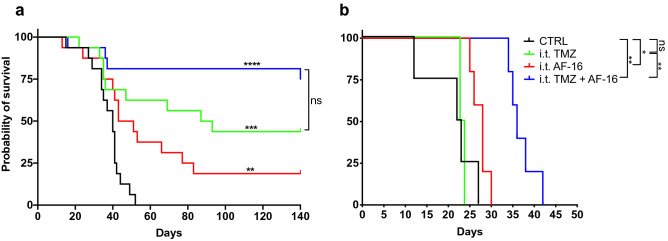
The effect of intratumoral AF16 on survival. Kaplan–Meier survival curves for C57BL/6 (**a**) and NOD-*Prkdc*^*scid*^ (**b**) mice orthotopically inoculated with the syngeneic murine glioblastoma cell line GL261 into the right hemisphere and later treated with AF16, TMZ, and AF16 + TMZ solutions in CED pumps. In (**a**), untreated C57BL/6 mice (black curve, n = 14) are compared with TMZ alone (green curve, n = 16, *p* < 0.001 ***), AF16 alone (red curve, n = 16, *p* < 0.01 **), and AF16 with TMZ (blue curve, n = 16, *p* < 0.0001 ****). TMZ alone compared to AF16 with TMZ showed *p* = 0.085. In (**b**), untreated NOD-*Prkdc*^*scid*^ mice (black curve, n = 4) are compared with TMZ alone (green curve, n = 4, *p* = 0.774), AF16 alone (red curve, n = 5, *p* = 0.035 *) and AF16 with TMZ (blue curve, n = 5, *p* < 0.01 **). TMZ alone compared to AF16 with TMZ showed *p* < 0.01 **. All log rank tests.

After intratumoral administration, TMZ (n = 16), AF16 (n = 16) and the combination AF16 + TMZ (n = 16) increased the overall survival (OS) of C57BL/6 mice with GL261 gliomas compared to control animals (n = 16) without any treatment (survival proportions controls 0/16, AF16 3/16, TMZ 7/16 and AF16 + TMZ 12/16; log-rank test *p* values against controls AF16 = 0.0048, TMZ = 0.0007 and AF16 + TMZ =  < 0.0001, Fig. 1a). When we subjected NOD-*Prkdc*^*scid*^ mice to the same therapy, we recorded prolonged survival in AF16 (n = 5) and AF16 + TMZ (n = 5) but not in the TMZ (n = 4) group (log-rank test *p* values against controls AF16 = 0.0353, TMZ = 0.774 and AF16 + TMZ = 0.0027, Fig. 1b). Furthermore, in this immunocompromised mouse model, there was a significant improvement of survival in the AF16 + TMZ group compared to only TMZ (log-rank test *p* value = 0.0039, Fig. 1b). Neither of the treatments resulted in cure in the NOD-*Prkdc*^*scid*^ mice.

### AF16 in combination with TMZ significantly decreased the size of murine GL261 gliomas

**Figure 2:**
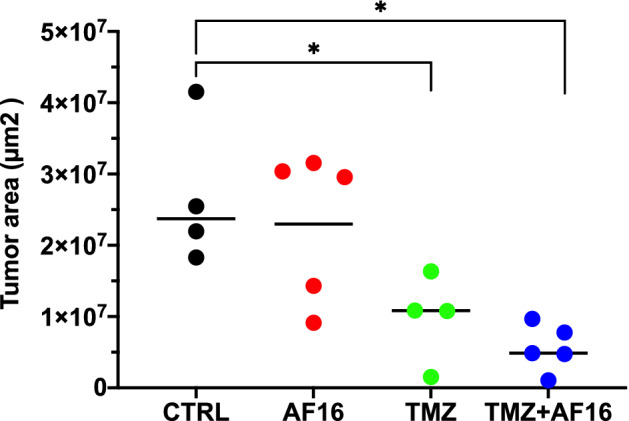
AF16 + TMZ combination and TMZ alone decrease tumor area in GL261-bearing C57BL/6 mice. When the first animal showed tumor symptoms, the whole cohort was sacrificed and tumor area was analyzed in brain sections. TMZ + AF16 compared to controls *p* = 0.016 *, TMZ alone compared to controls *p* = 0.029 *; both nonparametric Mann–Whitney U tests. Values are shown in µm^2^.

A separate histological in vivo study was conducted to elucidate the effect of AF16-based therapy on the tumor size and infiltration patterns in the TME of the GL261 malignant glioma. All mice were sacrificed on day 27 upon the appearance of symptoms in the first individual. Overall, we recorded a significant decrease of tumor surface area in the AF16 + TMZ group (n = 5, nonparametric Mann Whitney U-test, *p* = 0.0159) and the TMZ group (n = 4, *p* = 0.0286) compared to controls (n = 4). Group comparisons of controls against only AF16 (n = 5) or TMZ against TMZ + AF16 did not show statistical significance (Fig. 2).

### AF16 inhibits the TMZ-induced expansion of intratumoral macrophages and CD8^+^ T cells and increases galectin-3 and pNKCC-1 in the tumor microenvironment of murine GL261 gliomas

**Figure 3:**
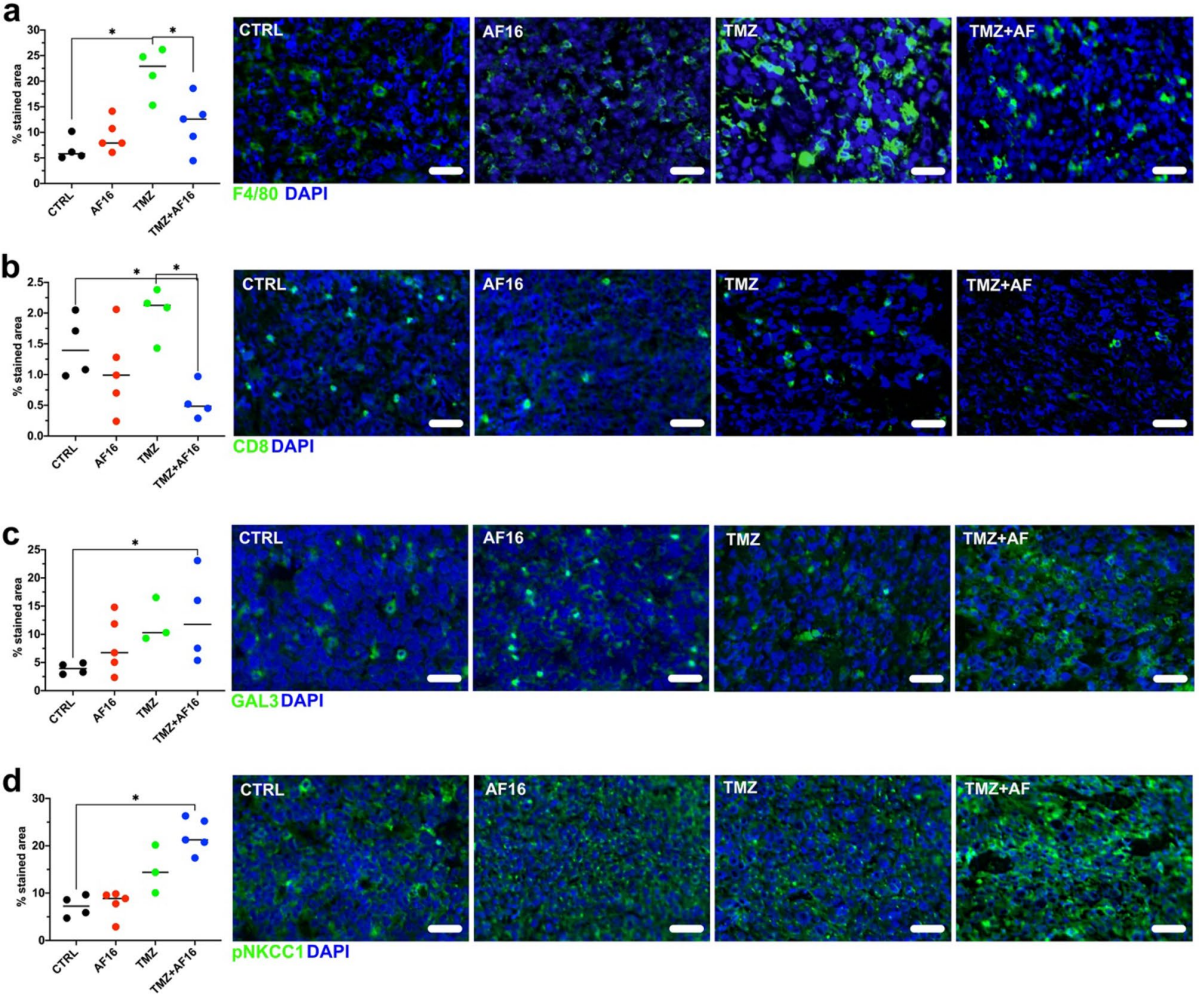
Changes in expression of immune cell surface markers and immune-related molecules caused by AF16 and TMZ treatment compared to untreated animals. Representation of immunohistochemical staining of brain sections of GL261-bearing mice shows an increase of F4/80^+^ macrophages after TMZ treatment was abolished by addition of AF16 (TMZ compared to TMZ + AF16 *p* = 0.032 *) (**a**). A decrease in CD8^+^ T cells was recorded after the combination treatment with TMZ and AF16 compared to untreated animals and TMZ alone (CTRL compared to TMZ + AF16 *p* = 0.029 *; TMZ compared to TMZ + AF16 *p* = 0.029 *) (**b**). Increased expression of galectin-3 was seen after the combination treatment with TMZ and AF16 compared to untreated animals (CTRL compared to TMZ + AF16 *p* = 0.029 *) (**c**). Similarly, the combination of TMZ and AF16 also increased the expression of pNKCC1 compared to untreated animals (CTRL compared to TMZ + AF16 *p* = 0.002 **) (**d**). Representative pictures are shown for each immunohistochemical staining, values are shown in percent of total tumor area. All tests are nonparametric Mann–Whitney U tests, scale bars correspond to 50 µm.

For this histological experiment, we used brain tissue from a different cohort of mice where treatment was postponed assuring sufficient tumor area for analysis. Upon staining of the brain tissue, we recorded a significant increase in the tumor-infiltrating F4/80^+^ macrophage population in the TMZ group compared to the controls, while this increase was inhibited by combined AF16 + TMZ (Mann–Whitney U test, TMZ v control *p* = 0.029, TMZ v AF16 + TMZ *p* = 0.032, Fig. 3a). AF16 by itself did not induce a change in the F4/80^+^ cells intratumorally. In a similar way to F4/80, CD8^+^ cells were decreased by the combination of AF16 + TMZ compared to controls and TMZ alone (*p* = 0.029, Fig. 3b). After further staining for galectin-3, implicated in glioma progression, as well as in membrane damage and other cellular processes, we observed a significant increase of galectin-3 staining after AF16 + TMZ compared to controls (*p* = 0.029, Fig. 3c). A similar increase of pNKCC-1, an activated ion pump essential for cell electrolyte homeostasis, was also recorded in the AF16 + TMZ group compared to controls (*p* = 0.002, Fig. 3d). Staining using the M2 macrophage markers CD206 and COX-2, as well as the M1 macrophage/dendritic cell markers CD11c and MHC II did not show any significant differences between treatments (Supplementary Fig. 1).

### AF16 drives the secretome of murine M0 macrophages towards the M1 macrophage profile

**Figure 4:**
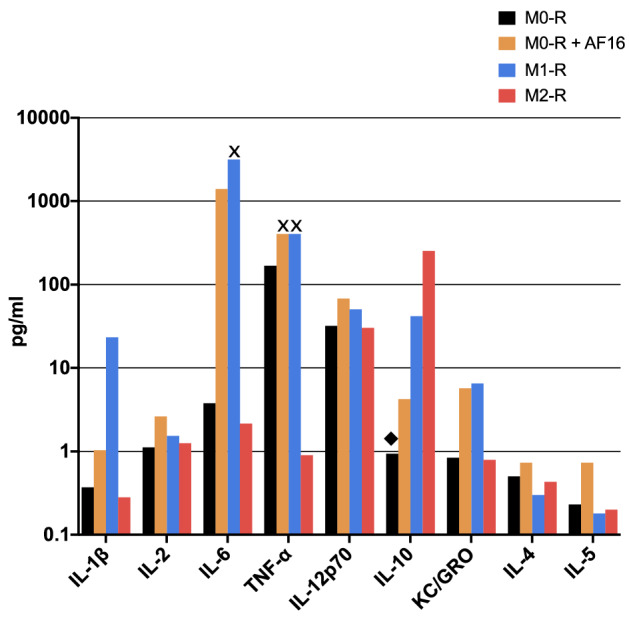
Overview of inflammation-related cytokines upregulated by AF16 treatment of RAW264.7 cells (M0-R). AF16 treatment upregulated all shown factors. Values from untreated cells (black columns) are shown next to AF16-treated cells (orange columns), as well as M1 and M2 differentiated macrophages (blue and red columns, respectively). Values marked with ◆ and **x** indicate where the original values were replaced by the lowest limit of detection or upper limit of quantification, respectively. Values are shown in pg/ml on a logarithmic scale.

Naïve RAW264.7 murine M0 macrophages were incubated with 2000 µg/ml AF16 to investigate the immunomodulatory properties of the molecule. This was compared with untreated RAW cells (M0-R) and RAW cells differentiated into M1 and M2 phenotypes. AF16 increased the majority of measured cytokines, including IL1β, IL2, IL6, TNFα, IL12p70, IL10 and KC/GRO to levels similar to M1 macrophages while their levels were reduced or unchanged in M2 macrophages, except for IL10 (Fig. 4). The analysis of collected supernatants showed that the M1 and M2 differentiation was successful based on the secretion of phenotype-specific cytokines compared to M0 macrophages (IL1β, IL6, TNFα increased in M1 and decreased in M2 cells; IL-10 increased substantially in M2 cells, Fig. 4).

### AF16 modulates the secretion of proinflammatory cytokines by human M0 macrophages

**Figure 5:**
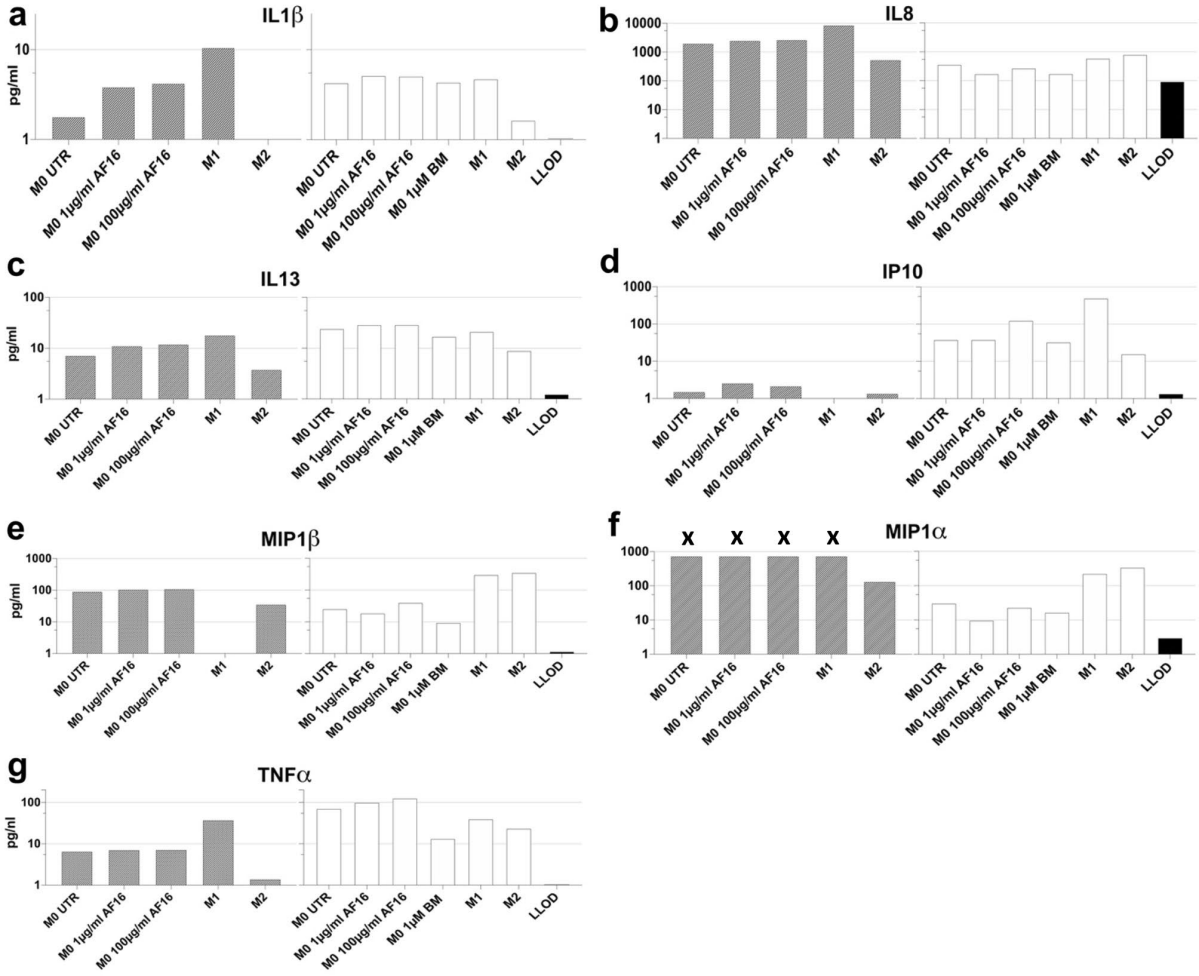
Overview of the inflammation-related factors secreted by human macrophages in response to AF16 treatment. THP-1 cells and primary human monocytes were differentiated into M0 macrophages (THP-1 derived M0, grey columns and primary mono-derived M0, white columns, respectively) and treated with 1 µg/ml and 100 µg/ml AF16 or 1 µM betamethasone (BM), or into M1 and M2 macrophages (M1, M2 respectively). Other M0 cells were left untreated (M0 UTR). The levels of IL1β (**a**), IL13 (**c**), IP10 (**d**) and TNFα (**g**) were upregulated in M0-T and M0-P cells after AF16 treatment, while IL8 (**b**), MIP1α (**f**), MIP1β (**e**) were upregulated in M0-T and downregulated in M0-P by AF16. Levels of MIP1α in THP-1 derived M0 and M1 cells were replaced by upper limit of quantification (marked with **x**). Values are shown in pg/ml on a logarithmic scale.

To investigate the effect of AF16 on cell line-derived and primary human macrophages, we treated cultures of M0 macrophages derived from the THP-1 cell line, as well as differentiated from primary human monocytes (see “Materials and methods” section) with 1 µg/ml and 100 µg/ml AF16 or 1 µM betamethasone as a control for inhibition of macrophage-derived inflammation. M0 macrophages were fully differentiated into M1 and M2 cells as controls for macrophage activation. In particular, an increase in IL1β from M0 macrophages after AF16 mirrors the increase in IL1β in M1 macrophages. M2 macrophages downregulated IL1β and betamethasone had negligible effect on IL1β levels in primary M0 macrophages (Fig. 5a). Furthermore, AF16 upregulated IL8 in supernatants of THP-1-derived macrophages but downregulated IL8 in primary macrophages while the rest of the cells upregulated IL8. Betamethasone downregulated IL8 from primary M0 macrophages (Fig. 5b). IL13 was upregulated in M0 macrophages after AF16, as well as in THP-1-derived M1 cells and downregulated in the rest of the cells and after betamethasone (Fig. 5c). IP10 was likewise upregulated in M0 macrophages and primary M1 cells after AF16 while M2 cells downregulated IP10. IP10 was undetectable in M1-T cells and BM had no effect on its production in primary M0 cells (Fig. 5d). TNFα was upregulated in M0 and primary M1 macrophages after AF16, downregulated in the other cell types and after betamethasone (Fig. 5g). MIP1β was upregulated by AF16 in THP-1-derived M0 macrophages, downregulated in primary M0 macrophages with levels undetectable in THP-1-derived M1 and decreased in M2-T cells. primary M1 and M2 cells upregulated MIP1β, while betamethasone treatment downregulated MIP-1β (Fig. 5e). MIP1α exceeded the upper detection limit in THP-1-derived M0 and M1 cells while it was downregulated in primary M0 cells both after AF16 and betamethasone. Both primary M1 and M2 cells increased MIP1α (Fig. 5f). In summary, AF-16 treatment increased the expression of IL1β, IL8, IL13, IP10, MIP1β and TNFα in the supernatants of primary and THP-1-derived macrophages.

### AF16 modulates the secretion of inflammatory proteins in cultured primary human glioblastoma cells

**Figure 6:**
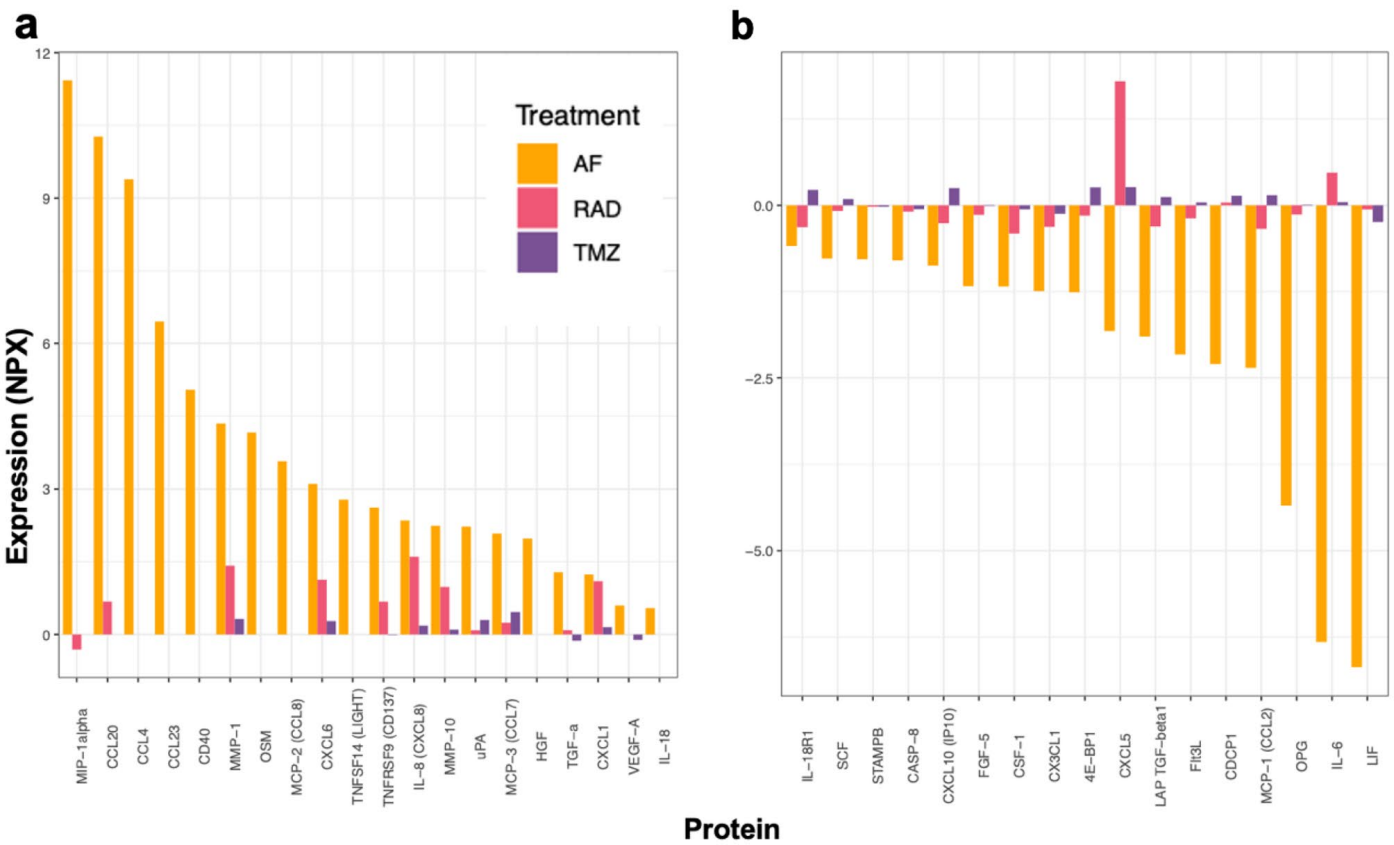
Changes in expression of proteins in cultured primary human GBM cell supernatants after AF16 treatment. Radiation by 20 Gy and treatment with 100 µM TMZ were used for comparison of immunomodulation by conventional treatment. Compared to untreated cells, proteins upregulated by 20 µg/ml AF16 are CCL3 (MIP1α), CCL4 (MIP1β), CCL7 (MCP3), CCL8 (MCP2), CCL20 (MIP3α), CCL23 (MIP3), CD40, MMP-1, OSM, CXCL6, TNFSF14, TNFRSF9, IL-8, MMP-10, uPA, TGFα and CXCL1 in panel (**a**). Proteins downregulated by 20 µg/ml are AF16 are LIF, IL-6, OPG, CCL2 (MCP1), CDCP1, Flt3L, LAP-TGFβ, CXCL5, 4E-BP1, CX3CL1, CSF1 (M-CSF), FGF5 and CXCL10 (IP10) in panel (**b**). AF16 caused more pronounced changes than radiation and TMZ in levels of all analyzed factors. Values are shown in normalized protein expression (NPX).

We wanted to investigate the immunomodulatory capacity of AF16 on primary human glioblastoma cells. AF16-treated cells were compared with cells either exposed to cytotoxic agents (irradiation or TMZ) or untreated cells and secreted proteins in cell culture supernatants were analyzed using the Olink® ProSeek Multiplex Inflammation I panel. Factors up-regulated by AF16 treatment were CCL3, CCL4, CCL7, CCL8, CCL20, CCL23, CD40, MMP-1, OSM, CXCL6, TNFSF14, TNFRSF9, IL-8, MMP-10, uPA, TGF-a and CXCL1 (Fig. 6a). The majority of these factors were also upregulated by irradiation. Proteins decreased by AF16 were LIF, IL-6, OPG, CCL2, CDCP1, Flt3L, LAP-TGF-β, CXCL5, 4E-BP1, CX3CL1, CSF1, FGF5 and CXCL10 (Fig. 6b). Most of these proteins were also inhibited by irradiation. TMZ treatment modulated most factors by < 0.5 NPX.

### Confirmation of AF16-modulated proteins in glioblastomas and astrocytoma tumor tissue

We further went on to investigate if the proteins modulated by AF16 were also expressed in tissue lysates of brain tumor tissue. We compared glioblastoma tissue (n = 3) with tissue from lower-grade brain tumors (astrocytomas grade II, n = 3) (Supplementary Fig. 2a,b). We also assessed the presence of AF16-modulated factors at the mRNA level in glioblastomas and astrocytomas included in the TCGA database. There, genes with a higher expression in glioblastoma compared to astrocytoma were VEGFA, 4EBP1, CCL2, PLAU (uPA), CXCL8 (IL8), CXCL10 (IP10), CASP8, HGF, LIF, CD40, CDCp1, TNFRSF11B (OPG), CD274 (PD-L1), IL6, CXCL1, CCL20 (MIP3α), CCL8 (MCP2), CD244, MMP1, TNFRSF9, CXCL6, MMP10, FGF5). Genes that show higher expression in astrocytoma compared to glioblastoma were CX3CL1, CSF1 (MCSF), CCL3 (MIP1α), CCL4 (MIP1β), TGFα, FLT3LG, CXCL5, CCL19 while KITLG (SCF) and NGF were equally expressed in the two tumor types (Supplementary Fig. 2c).

## Discussion

In this paper, we investigated the role of AF16 as an adjuvant therapeutic agent in the context of glioblastoma TME, and its effect on the function of macrophages. Previous reports show that blockade of AF16 potentiates the immune response from T cells and exogenous administration of AF16 decreases inflammation in the gut tissue and in immune encephalitis in rats^22,24,25,29^ but not in mice, despite treated animals having higher survival rates^37^. Others have shown that the parental molecule, antisecretory factor (AF), induced in mice by diet of specially processed cereals (SPC’s), exerts direct antitumor effects with increased tumor apoptosis and reduced proliferation in a human xenograft glioblastoma model^30^. Moreover, SPC diet reduced the interstitial fluid pressure inside the tumor and increased doxorubicin and erlotinib uptake into tumor tissue^30^. Additionally, administration of AF16 decreased interstitial pressure in solid tumors^28^, as well as intracranial pressure and edema following brain injury^26,27^.

It would be pertinent to break the chronic immunosuppression in the glioblastoma microenvironment that contains large numbers of tumor-associated macrophages^38–40^. Our results show that AF16 cured mice both alone and synergistically with TMZ in the immunocompetent mouse glioblastoma model, while also decreasing tumor size. Macrophages, microglia and dendritic cells are tentatively the most sensitive to AF16 as suggested by the increased survival after both AF16 alone and synergistically with TMZ in the immunocompromised NOD-*Prkdc*^*scid*^ mouse strain lacking B and T lymphocytes and displaying hypogammaglobulinemia^41^. Given that there were no treatment-related changes in tumor infiltration by CD206^+^ M2 macrophages nor CD11c^+^/MHC II^+^ M1 macrophages/dendritic cells, we postulate that the F4/80^+^ population increased by TMZ consists of naïve M0 TAMs and microglia, and that addition of AF16 to TMZ diminished this population, possibly by activation-induced cell death^42^. Moreover, surface marker expression should be combined with functional tests, such as Mesoscale and Olink, due to the promiscuous expression of markers on different cell types, including tumor cells themselves. The results from the in vitro treatment of human and murine M0 macrophages suggest that AF16 induces changes in the M0 secretome that mimic the M1 profile with several proinflammatory cytokines upregulated (IL-1β, IL-8, TNF-α, IP-10, MIP-1α and MIP-1β in human; and IL-1β, IL-2, IL-6, TNF-α and IL-12p70 in murine macrophages). Such AF16-induced secretion of proinflammatory cytokines by the increased M0 intratumoral macrophage population together with the effect of TMZ could be responsible for the in vivo survival benefit, tumor size decrease, and activation-induced cell death of the F4/80^+^macrophages/microglia and the CD8^+^ T cells^42,43^. We recorded discrepancies in levels of factors between THP-1-derived cells and primary human cells. This has been reported previously^44,45^ and it reflects the inherent differences of an immortalized tumor cell line as a simplified model of human monocytic-like cells, as well as the diversity in immune responses between individuals.

Unexpectedly, local administration of AF16 combined with TMZ led to an increase in galectin-3 and pNKCC-1 expression intratumorally compared to untreated animals. Galectin-3 is an immunosuppressive molecule in the tumor microenvironment, and it is expressed abundantly in human macrophages and tumor cells under stress, i.e. cytostatic therapy^34,46–48^. Under physiologic conditions, galectin-3 is associated with lysosomal membrane damage and autophagy, among other effects^33^. The increased expression of galectin-3 detected after AF16 and TMZ treatment could be explained by the cytotoxic effect of this therapy causing membrane damage and increased autophagy as part of regulated death of tumor cells and senescent immune cells. NKCC-1 and its active phosphorylated form (pNKCC-1) have recently been implicated to play an important role in macrophage activation^49,50^ as well as in glioma invasion^51^. Ilkhanizadeh et al. reported that diet of specially processed cereals, which induce host production of AF, decreased the expression of pNKCC-1 on human glioblastoma cells growing in immune-depleted mice^30^. There is, however, no mention of the effect on the tumor-infiltrating macrophages. Given that we demonstrated that AF16 caused a surge in proinflammatory cytokines in human and murine macrophages, this activation of the M0 population can be the cause of the pNKCC-1 increase we saw in mice receiving the combination of AF16 and TMZ. After staining of brain tissue for COX-2, a key enzyme in the production of prostaglandins, we did not see a difference between treatment groups, implying that prostaglandin signaling is not affected by AF16.

In addition to macrophage modulation, we wanted to elucidate the AF16-induced effect on the inflammatory secretome of primary human glioblastoma cells. Among the proteins which expression was increased by AF16 on human glioblastoma cells were proinflammatory chemoattractants of granulocytes, T cells and monocytes/macrophages (CCL3, CCL4, CCL7, CCL8, CCL20), T cell-stimulatory signals (CD40, TNFSF14, TNFrSF9), as well as metalloproteinases and other enzymes (MMP-1, MMP-10, uPA) or other molecules (IL8, TGFα). Several of these factors have been associated with glioblastoma growth and progression^52^, while their expression was also increased by irradiation (MMP1, CXCL6, IL8, MMP10, CXCL1). The proteins expressed by human glioblastoma cells that were decreased by AF16 have in the majority of cases been associated with lower T cell and higher myeloid cell tumor infiltration (LIF, IL6, CSF1), glioblastoma progression and poor prognosis (OPG, CDCP1, CXCL5, 4E-BP1, CX3CL1, FGF5, LAP-TGFβ)^53–55^, while some factors (CCL2, Flt3L, CXCL10) were described as proinflammatory and potentially tumor-suppressive^56^. Irradiation had negligible effect on factors in the “AF16-decreased” group, except on CXCL5, which it increased. Overall, the human glioblastoma secretome analysis of AF16-induced changes showed a complex picture of proinflammatory and tumor-promoting factors that were modulated by AF16, with several tumor-promoting factors decreased, which could contribute to the favorable survival in vivo. Of note, TMZ treatment induced only minor changes in the inflammatory secretome of human glioblastoma cells in our experiment. Furthermore, analysis of fresh-frozen surgical samples of human glioblastoma and Grade II astrocytoma reveals that several proteins with higher expression in glioblastoma compared to astrocytoma were downregulated by AF16 treatment (4EBP1, CCL2, CXCL10, LIF, CD40, OPG, IL6, FGF5) while correspondingly, some factors with higher expression in astrocytoma compared to glioblastoma were also upregulated by AF16 in glioblastoma (CCL3, CCL4, TGFα). This could be evidence that AF16 modifies the TME of glioblastoma to become less pro-malignant while acknowledging that several glioblastoma-associated proteins were upregulated by AF16 treatment (uPA, IL8, etc.).

In summary, we propose AF16 as a novel immunomodulatory molecule that can cure immunocompetent mice and prolong overall survival in immunodeficient mice with high-grade glioma. It also stimulates the production of proinflammatory cytokines in human and murine M0 macrophages. In this regard, AF16 mimicked the cytokine pattern induced by M1 differentiation and exerted opposite effect than betamethasone on several factors. Moreover, AF16 modulated several proteins associated with glioblastoma progression and inflammation, secreted by primary human glioblastoma cells. Our trial study is limited by the low number of human cell and tissue donors, as well as lack of deeper mechanistic investigations. With further research in this area warranted, AF16 as an active moiety of the ubiquitous antisecretory factor protein could be an attractive candidate for therapy without known side effects^31^ in conditions with a dysregulated immune response, i.e. glioblastoma.

## Materials and methods

### Cell medium and culture, cytokines and antibodies

Unless otherwise stated, all culturing media used were RPMI 1640 supplemented with 2 mM L-Glutamine, 1 mM sodium pyruvate, 10 mM HEPES, 50 μg/ml gentamicin (Invitrogen AB, Sweden) and 10% FBS (Biochrom AB, Germany), henceforth referred to as R10 medium. All cells were kept in a humidified incubator at 37ºC in 5% CO_2_ atmosphere. Dividing cells were passaged regularly to avoid overgrowth and were kept in culture for less than 20 passages before analysis. All handling of cells occurred under sterile conditions. The following cytokines were used for macrophage differentiation cocktails: human IFN-γ (Gibco, Life Technologies, Sweden), LPS (Sigma-Aldrich Sweden AB, Sweden), recombinant human IL-4, human IL-6, human M-CSF, human GM-CSF (all Miltenyi Biotec, Sweden), PMA, mouse IL-10 (both Sigma-Aldrich Sweden AB, Sweden). The following antibodies were used for staining: mouse anti-human CD14 (BD Pharmigen™, BD Biosciences, USA; Clone M5E2, Cat. # 555397), rat anti galectin-3 (Invitrogen, ThermoFisher Scientific, Stockholm, Sweden; clone eBioM3/38 (M3/38), Cat. # 14-5301-82), rabbit anti phospho-NKCC1 polyclonal antibody Thr212/Thr217 (EMD Millipore Corporation, USA; Lot # 3474199, Cat. # ABS1004), PE hamster anti-mouse CD11c (BD Pharmigen™, BD Biosciences, USA; Lot # 0000049812, Cat. # 553802), FITC rat anti-mouse CD8α (BD Pharmigen™, BD Biosciences, USA; Lot # 4073826, Cat. # 553030), rabbit polyclonal anti-Mannose receptor/CD206 (Abcam, UK; Cat. # ab64693, Lot # GR254714-1), rabbit polyclonal anti-COX2 (Abcam, UK; Cat. # ab15191, Lot GR3267180-3), rat anti-mouse F4/80 (AbD Serotec®, Nordic BioSite, Stockholm, Sweden; Cat. # MCA497), mouse biotin anti-mouse MHC II (BD Pharmigen™, BD Biosciences, USA, Lot # M064787, Cat. # 553550), goat anti-rat IgG AlexaFluor™ 488, goat anti-rabbit IgG AlexaFluor™ 488, Streptavidin AlexaFluor™ 488 (Invitrogen, ThermoFisher Scientific, Stockholm, Sweden; Cat. # A-11006; Cat. # A-11034; Cat. # S-32354).

### Convection-enhanced delivery (CED) of AF16 in mouse glioblastoma model in immunocompetent and immunodeficient animals

All animal experiments were conducted in accordance with the ARRIVE guidelines. Animal procedures were approved by the Ethical Committee for Animal Research in Lund-Malmö, permit numbers M151–15 and 14006/2019, and were performed in accordance with the practices of the Swedish Board of Animal and European Union Animal Rights and Ethics Directives. Mice were observed daily, and individuals were euthanized immediately by cervical dislocation when neurological signs of tumor growth occurred (inactivity, tremors, hunched posture, and weight loss). The primary endpoint was overall survival (OS) and animals that reached day 150 after tumor inoculation were considered cured. C57BL/6 mice (8–10 weeks-old females, *n* = 62, purchased from Taconic Bioscience A/S, Denmark) and NOD scid mice (8–10 weeks-old females NOD-*Prkdc*^*scid*^, *n* = 18, bred at the BMC in-house breeding core facility, Lund University, Sweden) and were each stereotactically injected with cells of the mouse glioma cell line GL261 (5000 cells/3 µl injection volume, cells kindly provided by Dr. G Safrany, “Frédéric Joliot-Curie” NRIRR, Hungary) into the right cerebral hemisphere under general anesthesia, according to an established protocol^57,58^. Briefly, on day 7 post-injection, pumps for intratumoral delivery (3-day mini-osmotic pumps Alzet® model 1003D, fill volume 100 μl, pumping rate 1 μl/h (DURECT™ Corporation, USA)) were filled with 300 µg/72 µl AF16 solution (NOD-*Prkdc*^scid^ *n* = 5, C57BL/6 *n* = 16) or 180 mg/72 µl temozolomide (TMZ; Temodal 2,5 mg/ml [Merck Sharp & Dohme, Sweden]) solution (NOD-*Prkdc*^scid^ *n* = 4, C57BL/6 *n* = 16) or the combination of AF16 300 µg and TMZ 180 mg/72 µl (NOD-*Prkdc*^scid^ *n* = 5, C57BL/6 *n* = 16) and implanted subcutaneously into anesthetized mice. The catheter was delivering the treatment intratumorally in the cerebrum through the original burr hole. The skin incision was closed using metal clips. After the designated working time, the pumps were removed under general anesthesia. One group (NOD-*Prkdc*^scid^ n = 4, C57BL/6 *n* = 14) did not receive any pumps or therapy. Mice were kept under standard conditions with constant access to food and water according to local animal facility guidelines.

### Histological evaluation of AF16-induced changes in the mouse glioma microenvironment

C57BL/6 mice (8–10 weeks-old females, *n* = 20, purchased from Taconic Bioscience A/S, Denmark) were each stereotactically injected with the GL261 glioma cell line on day 12 and treated with AF16 (*n* = 5), TMZ (*n* = 5) and the combination of both (*n* = 5) as described above with one group left without treatment as a control (*n* = 4). One animal died immediately after tumor cell inoculation likely due to surgical complications and was therefore excluded from analysis. As soon as the first animal developed neurological symptoms, the whole cohort was euthanized and fresh cerebra were harvested. Immediately after harvest, the brain tissue was frozen and fixed in cooled isopentane (− 55 °C, VWR International AB) and kept thereafter at − 80 °C until further analysis. Due to small tumor volumes, one animal each in TMZ and TMZ + AF16 groups could not be analyzed for all markers. Before staining, brain tissue was sectioned into 6 µm-thick sections on a cryostat (Leica, Germany). Then, sections were fixed with acetone for 10 min at room temperature, rehydrated with PBS, blocked for 20 min with 5% goat serum (Jackson ImmunoResearch, USA) and stained with antibodies before being mounted in DAPI-containing mounting medium (ProLong™ Gold antifade, Invitrogen). Tumor area was measured in the largest diameter in serial sections from different treatment groups. Areas stained with F4/80, CD206, CD11c, MHC II, CD8, COX-2, galectin-3 and pNKCC-1 were measured with the cellSens Dimension software on the fluorescent microscope BX-53 (both Olympus LRI Instrument, AB). Negative control sections were used for validation where the primary antibody was omitted.

### Mapping of inflammation-associated secretome of primary human brain tumors

Brain tumor cells and tissue were obtained from Södra sjukvårdsregionens CNS tumor biobank Dnr. 2018/37 under ethical permit 2008/642, approved by the Local Ethical Review Board in Lund. Measurements of proteins was performed in supernatants from cultured human primary glioblastoma cells treated with irradiation (20 Gy), 20 µg/ml AF16 or 100 µM TMZ for 24 h. Untreated glioblastoma cells were used as controls. Glioblastoma cells were seeded into a 24-well plate at 100,000 cells/well in 1 ml R10 cell culture medium. In addition, proteins were also assessed in frozen samples of glioblastoma (n = 3) and astrocytoma (n = 3) tissues. Frozen tissues (100 mg) were homogenized in 1 ml MSD Tris Lysis Buffer (MesoScale Discovery, Rockville, MD, USA) and incubated on ice for 20 min. The lysates were centrifuged for 10 min at 2000 g. Supernatants from the lysates and from cultured cells were collected and stored at − 80 °C until subsequent analysis. Proteins from cultured tumor cells and tissue lysates were prepared for and analyzed using the ProSeek multiplex Inflammation I panel from Olink proteomics according to manufacturer´s instructions (Olink Biosciences, Uppsala, Sweden). Values were expressed as normalized protein expression (NPX) where one-NPX difference equals a doubling of the protein concentration. Values below lowest limit of detection (LOD) were set to the LOD value. Values above the upper limit of quantification (ULOQ) were set to the value of ULOQ. Differences in protein expression of cell culture supernatants were calculated by subtracting the value of treated cells with the value of untreated cells.

### Maturation of human M0, M1 and M2 macrophages from THP-1 cells

Human monocytic cell line THP-1 (ATCC TIB-202; LGC Standards, UK) were cultured in R10 medium. To obtain M0 macrophages, THP-1 cells were incubated with 62 ng/ml PMA in R10 for 72 h. Then, the PMA-containing medium was discarded and cells were washed with fresh R10 twice, each cycle lasting 4 h. These cells are referred to as M0-T macrophages. The M0-T macrophages were further polarized into M1-T and M2-T cells with the appropriate cytokine cocktails for another 24 h (100 ng/ml LPS and 20 ng/ml IFN-γ for M1; 20 ng/ml rhIL-4 for M2a).

### Maturation of murine M1 and M2 macrophages from RAW 264.7 cells

The RAW264.7 murine macrophage cell line (ATCC TIB-71, LGC Standards, UK) were cultured in R10 medium. RAW264.7 cells correspond to M0 stage of macrophage maturation (M0-R) and they were incubated for 24 h with 100 ng/ml concentration of LPS and 20 ng/ml concentration of murine IFN-γ to obtain M1 macrophages (M1-R) or 20 ng/ml concentration of murine IL-10 to obtain M2 macrophages (M2-R).

### Maturation of M0, M1 and M2 macrophages from primary human monocytes

Primary human macrophages were differentiated by a slight modification of the protocol described by Zarif et al.^59^. Briefly, peripheral blood mononuclear cells (PBMCs) from healthy donors (n = 2) were purchased from the local blood donation bank and separated by density centrifugation of the buffy coat on Ficoll-Paque™ (GE Healthcare Life Sciences, Sweden). Next, highly purified classical monocytes were obtained with the Classical Monocytes MACS Isolation Kit (Miltenyi Biotec, Germany) following the manufacturer’s instructions. After purification, classical monocytes were incubated with 20 ng/ml M-CSF for 6 days to obtain M0 macrophages with medium changed on Day 1 and Day 3. To obtain finally differentiated M1 and M2 cells (M1, M2), M0 cells were washed with R10 at Day 6 and cultured for another 24 h in either a M1 cytokine cocktail (20 ng/ml GM-CSF, 20 ng/ml IFN-γ, 20 ng/ml IL-6 and 20 ng/ml LPS) or a M2 cytokine cocktail (20 ng/ml M-CSF, 20 ng/ml IL-4, 20 ng/ml IL-6), respectively.

### MesoScale discovery multiplex plate assay

Macrophages (M0) were incubated with 1 µg/ml and 100 µg/ml AF16 or 1 µM betamethasone for 24 h. Murine M0 macrophages (M0-R) were incubated with 2000 µg/ml AF16 for 24 h. Supernatants were collected from culture plates under sterile conditions and centrifuged at 12,000 rpm for 10 min to separate any solid particles. The final supernatants were then removed and frozen at − 20 °C until analysis. The supernatants were analyzed in duplicates with MesoScale V-PLEX® (MesoScale Discovery, USA) human Proinflammatory Panel 1 (IFNγ, IL1β, IL2, IL4, IL6, IL8, IL10, IL12p70, IL13, TNFα), human Chemokine Panel 1 [Eotaxin, Eotaxin-3, IL8 (HA), IP10, MCP1, MCP4, MDC, MIP1α, MIP1β, TARC] and mouse Proinflammatory Panel 1 (IFNγ, IL1β, IL2, IL4, IL5, IL6, IL10, IL12p70, KC/GRO, TNFα) according to the manufacturer’s instructions. When analyzing data, only the range between Lower Level of Detection (LLOD) and Upper Level of Quantification (ULOQ) was considered and when values were below the LLOD or above the ULOQ, they were replaced by the LLOD/ULOQ values. In total, cells from two different human donors and one representative THP-1 experiment are shown. Data are shown in pg/ml on a logarithmic scale. Factors where change was induced in both donors are included (all raw data included in Supplementary Table 1).

### Confirmation of proteomics data with mRNA expression in the TCGA database

To validate previous results in a large brain tumor dataset, low grade glioma and glioblastoma level 3-preprocessed mRNA expression data were obtained from The Cancer Genome Atlas (TCGA) database at the GDS website: https://portal.gdc.cancer.gov/. The R/Bioconductor package, TCGAbiolinks, was used for database access.

### Data analysis

All data was analyzed and visualized using the Prism 8 Software (GraphPad, USA) or the free statistical software R (https://www.R-project.org/ ). Animal survival data were visualized with Kaplan–Meier curves and the treatment groups were compared with log rank tests with median survival. Non-parametric Mann–Whitney U-test was used to analyze the tumor area and immunohistochemical data from mouse brain sections.

## Supplementary Information

Supplementary Information.
