# Supplementary material for: Intratumoral administration of the antisecretory peptide AF16 cures murine gliomas and modulates macrophage functions
Source: Sci Rep. 2022 Mar 17;12:4609. doi: 10.1038/s41598-022-08618-x (PMC8930985; doi:10.1038/s41598-022-08618-x)
Supplement: Supplementary file 1 — Supplementary Information. [file 41598_2022_8618_MOESM1_ESM.docx]

Intratumoral administration of the antisecretory peptide AF16 cures murine gliomas and modulates macrophage functions

Jan Kopecky^1*^, Julio Enríquez Pérez^1^, Håkan Eriksson^3^, Edward Visse^1^, Peter Siesjö^1,2^, Anna Darabi^1^

^1^Glioma Immunotherapy Group, Division of Neurosurgery, Department of Clinical Sciences Lund, Faculty of Medicine, Lund University, Lund, Sweden

^2^Division of Neurosurgery, Department of Clinical Sciences Lund, Skåne University Hospital, Lund, Sweden

^3^Department of Biomedical Science, Faculty of Health and Society, Malmö University, Malmö, Sweden.

*Corresponding author: Jan Kopecky, Glioma Immunotherapy Group, Barngatan 4, 221 85 Lund, Sweden. E-mail address: [jan.kopecky@med.lu.se](mailto:jan.kopecky@med.lu.se)

**Supplementary Table S1**

a)

| pg/ml | primary human macrophages | AF16 1µg/ml | AF16 100µg/ml | BM | LLOD | ULOQ |
| --- | --- | --- | --- | --- | --- | --- |
| IFNγ | 2.74703989 | 3.02984772 | 3.02984772 | 1.19821497 | 0.37 | 938 |
| IL2 | 2.92795231 | 2.94657653 | 3.21068838 | 1.68877474 | 0.09 | 938 |
| IL4 | 0.53420248 | 0.5960723 | 0.58493323 | 0.27026762 | 0.02 | 158 |
| IL6 | 585.800668 | 1265.56798 | 1123.42256 | 5.37942933 | 0.06 | 488 |
| IL10 | 11.0586113 | 14.546428 | 18.5733994 | 5.9155025 | 0.04 | 233 |
| IL12p70 | 1.11996649 | 1.2687358 | 1.40641627 | 0.8055154 | 0.11 | 315 |

b)

| pg/ml | primary human macrophages | AF16 1µg/ml | AF16 100µg/ml | BM | LLOD | ULOQ |
| --- | --- | --- | --- | --- | --- | --- |
| Eotaxin | 9.42485265 | 0 | 7.39996968 | 3.19888634 | 3.26 | 1120 |
| Eotaxin3 | 0 | 0 | 0 | 0 | 1.77 | 3750 |
| MCP1 | 1510.71932 | 1388.96441 | 1542.0322 | 1117.59 | 0.09 | 375 |
| MCP4 | 27.8952175 | 24.4595397 | 41.2094152 | 14.8411252 | 1.69 | 469 |
| MDC | 4440.36625 | 1873.38136 | 3779.8592 | 2990.01614 | 1.22 | 7500 |
| TARC | 8.3928318 | 1.85164853 | 4.65298734 | 3.60438151 | 0.22 | 1120 |

c)

| pg/ml | THP-1 derived macrophages | AF16 1µg/ml | AF16 100µg/ml | LLOD | ULOQ |
| --- | --- | --- | --- | --- | --- |
| IFNγ | 0 | 0 | 0 | 0.2 | 938 |
| IL4 | 0 | 0 | 0 | 0.02 | 158 |
| IL6 | 0 | 0 | 0 | 0.06 | 488 |
| IL10 | 0 | 0 | 0 | 0.03 | 233 |

d)

| pg/ml | THP-1 derived macrophages | AF16 1µg/ml | AF16 100µg/ml | LLOD | ULOQ |
| --- | --- | --- | --- | --- | --- |
| Eotaxin | 0 | 0 | 0 | 3.26 | 1120 |
| Eotaxin3 | 0 | 0.33241 | 0.682454 | 1.77 | 3750 |
| MCP1 | 17.62728 | 19.00239 | 19.78361 | 0.09 | 375 |
| MCP4 | 2.439393 | 2.191827 | 2.843793 | 1.69 | 469 |
| TARC | 0 | 0 | 0.130725 | 0.22 | 1120 |

e)

| pg/ml | RAW264.7 cells | AF16 2000µg/ml | M1 | M2 | LLOD | ULOQ |
| --- | --- | --- | --- | --- | --- | --- |
| IFNγ | 0.1 | 0.2 | 172.68 | 0.08 | 0.04 | 570 |

**Supplementary Table 1.** Data on primary human inflammatory cytokines (a) and chemokines (b), THP-1 derived cytokines (c) and chemokines (d), and murine inflammatory cytokines (e) from cell culture supernatants analyzed by MesoScale Discovery Multiplex assays and not included in the main text. Treatment-induced change in these factors was either negligible or the values exceeded the reliable detection range. Values are shown in pg/ml.

**Supplementary Figure S1**

**Supplementary Figure 1. AF16 treatment doesn’t affect COX2, CD206, CD11c and MHC II in glioma sections.** There were no significant differences in the expression of COX2, CD206, CD11c or MHC II in the GL261 microenvironment. After staining brain and tumor tissue from GL261-bearing C57BL/6 mice for COX2 (a), CD206 (b) or CD11c (c) and MHC II (d), we recorded no significance in the difference between untreated and treated animals in these factors. Values shown in percent of stained area.

**Supplemenary Figure S2**
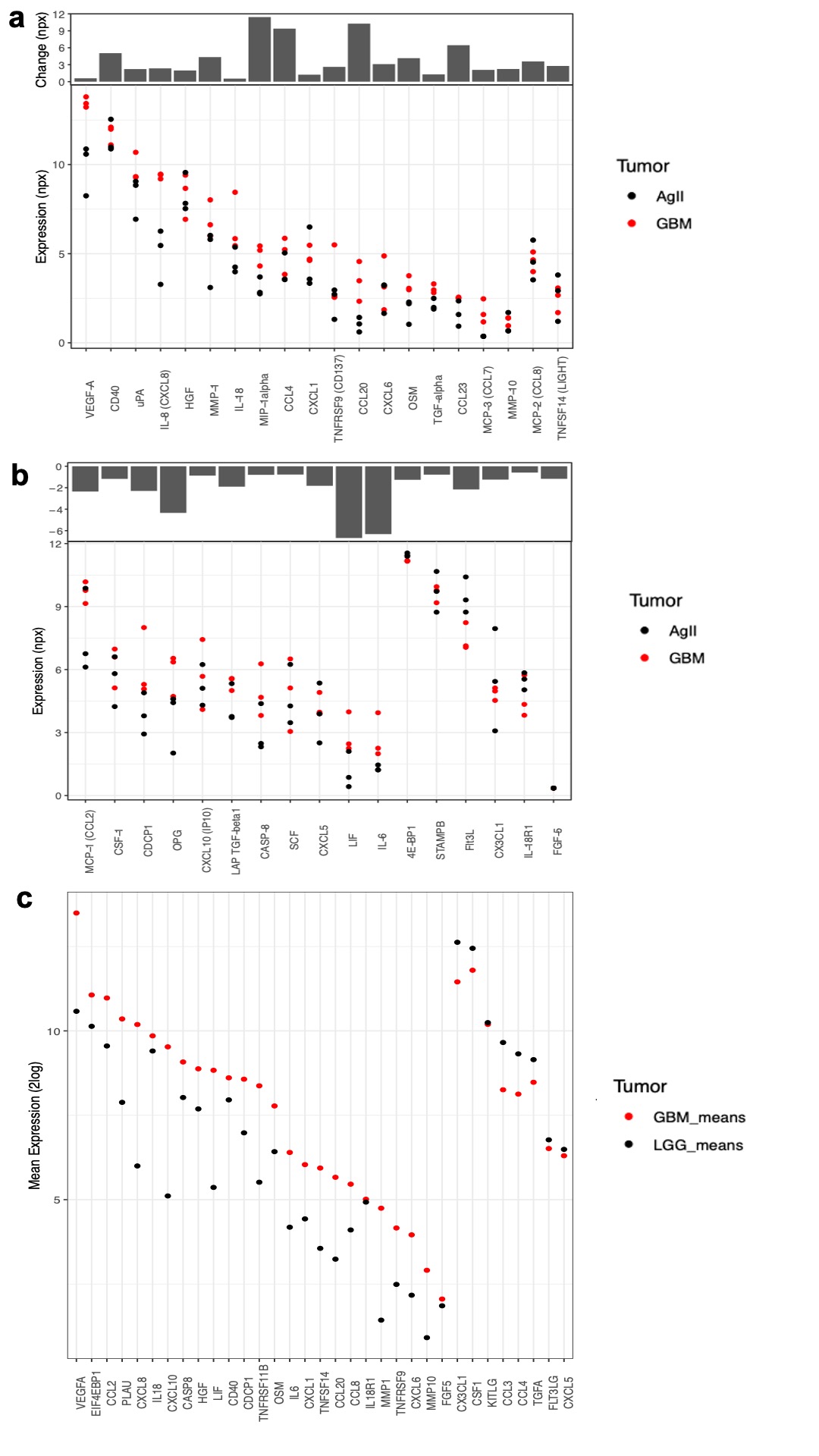


**Supplementary Figure 2.** A list of proteins differentially expressed in Grade II astrocytoma (LGG, black dots) and GBM (red dots). Protein expression analysis of human GBM and anaplastic astrocytoma tissue lysates shows the same proteins that were upregulated (**a**) or downregulated (**b**) by AF16 in cultured human GBM cells (upper panels) with differential expression between the tumor types (lower panels). The change in expression is in NPX units (see Materials and Methods). mRNA transcription analysis of human GBM samples in the TCGA database (**c**) shows proteins with higher levels in GBM than in LGG on the left, several of which were downregulated by AF16 in GBM (EIF4EBP1, CCL2, CXCL10, LIF, CD40, TNFRSF11b/OPG, IL6, FGF5). To the right side, there are proteins with higher levels in LGG than in GBM, some of which were upregulated by AF16 in GBM (CCL3, CCL4, TGFα). Mean expression difference is on a binary logarithmic scale.
